# The Replacement of the Teeth, with a Plan for Their Retention

**Published:** 1853-01

**Authors:** C. T. Cushman


### ARTICLE V.

### *The Replacement of the Teeth, with a Plan for their Retention.*

BY C. T. CUSHMAN., D. D. S.

THE violent accidents to which persons are daily subject, involving serious injuries of the mouth and teeth, render it injunctive upon dentists to be prepared for such emergencies, and to treat judiciously those cases which are liable to come under their care, at the shortest notice.

When the front teeth are dislodged from their sockets by any sudden·mechanical violence, and it is deemed advisable to replace them, there is no time to be lost in hunting up authorities for practice, or in deliberating about plan or precedent. It would be well, therefore, for the young practitioner, inexperienced in such cases, to be settled in policy beforehand, based on correct anatomical, physiological and pathological principles, and well versed in the history of homogeneous cases with which our professional literature is furnished.

That such dislocated teeth have been restored to their sockets and retained, in some cases, for many years, subserving their usual functions in a manner far superior to any artificial substitute, and without inconvenience or detriment to local or general health, is not problematical.

The remarkable success of the elder GARDETTE in the practice of luxating and replacing teeth is confirmatory of this. Among other numerous cases, he extracted a superior cuspid tooth, plugged it and replaced in its socket, where it soon got firm, and remained and did good service for about 18 years.*

Less than twenty years ago this practice was adopted and highly recommended by Dr. Bostwick.†

*Vide Phil. Med. Rec., v. xi, p. 104 or (Republished in) Am. Jour. D. S., v. x, p. 71.

†He says, I have lately been in the habit of destroying the nerve in preference to extracting the tooth, when the patient has but few teeth remaining, and none to spare, by starting it from its socket. To effect this object, I have entirely separated the gum from the tooth, and then, with the plain straight forceps, have removed the tooth sufficiently to separate the nerve from the jaw, which is done by the slightest movement. I then push it immediately back into its proper place, so quickly as to prevent any blood from insinuating itself between the roots of the teeth and the jaw-bone. The patient, in this case, must then bring his teeth firmly together, to keep the loosened one in its place, and must continue to do so for several days. In the course of a week, every disagreeable sensation in the tooth will cease, when it may be plugged and saved. I have performed this operation on the back teeth numerous times, with success. In some instances it has failed ; but those who have suffered the loss of nearly all their teeth, are willing to endure much pain, in preference to losing any more. It is always desirable to save a tooth which is in a salvable state, but more particularly when they have but few companions remaining to cheer their latter days. The easiest way of effecting this object is

Fitch, saw a man who had a dislocated tooth replaced, which remained firm for 30 years.*

The utility of the operation, judiciously performed, has been virtually demonstrated, to my own observation, in several cases which I have met with. Among the recent ones is the following:

CASE 1.—A healthy young mechanic, æt. about 30, of sanguine temperament, with firm gums, hard, sound teeth, regular and full denture, was holding a kind of chisel, weighing about eight ounces, with a pair of tongs, over an anvil on which an assistant was striking with a sledge hammer. A one sided blow glanced from the chisel and shot it with immense velocity directly against the mouth of the holder.

The inferior lip was severely cut horizontally; the superior incisors, right, central and lateral, were broken off at the neck. In the *inferior jaw*, on the right side, the crown of the cuspidatus was broken off about midway, an oblique fracture, leveled inwards; *the right lateral and central incisors, were completely ejected from their sockets.*

About one hour afterwards, they were replaced in their sockets, and very soon became firm and useful. This was six years ago; during which time they have "given no trouble." Their color is good, and one could scarcely distinguish any abnormal change in them in this respect.

The surrounding gum is firm and healthy, there is apparently no discharge of diseased secretions. The lateral is a little infirm; but I attribute the fact, to the accumulation of *tartar* upon it, which has been suffered to remain a long time—till quite hard.

Both the replaced teeth were about one-sixteenth of an inch above their proper altitude, this projection I filed away nearly to a level with the others.

It should be remembered, however, that in addition to the usual lymphatic effusion and thickening of the membranes in doubtless the best; and long experience has convinced us, that this is one of the best methods of effecting the object, and I think it decidedly to be preferred.—"*Family Dentist,*" pp. 84-5

*Dental Surgery, p. 185.

such cases, they had no antagonists during the six years. Consequently they were much more projected, probably, and more infirm than they otherwise would have been.

Although they were *not secured by ligatures, or other means*, when replaced, as, in my opinion, they certainly should have been, I still regard it as a remarkably favorable case. That these teeth will yet do good service for many years, and are altogether preferable to artificials, I have but the opinion affirmative. So sudden and forcible was the blow, and so heavy the missile, he did not experience much pain at the time of the accident; but considerable inflammation and soreness supervened.

Immediately after replacement, and for some weeks following, they were, I was informed, quite sensitive to pressure; but this gradually wore away, and they became quiet, unobtrusive, and *industrious* tenants of their natural habitations.

The roots of the superior central and lateral incisors, also of the inferior cuspidatus, had their pulps exposed by the fractures, which rendered it necessary to extirpate them with a probe; this was done soon after the accident. Subsequently the two former gave no trouble for nearly four years, when, from the habit of constantly probing them with a tooth-pick, and the ravages caused by decay, they became painful, and had to be extracted.

Where the crown of a healthy tooth is broken off suddenly, without much loosening of the root, I should advise the retention of the root, for the use of an artificial crown, or for mastication, or other purpose. In this, however, my judgment would be influenced by the temperament, general health, and particularly by the condition of the remaining teeth, gums, and secretions of the mouth.

Tomes mentions the case of a man who had the central incisors broken off by a play ball; the roots were "pivoted," (doweled,) and three years after, when the case was reported, "seemed perfectly free from disease."*

An instance of *failure* in restoring luxated teeth, which came under my observation, is exemplified in the following:

*Lectures on Dental Physiology and Surgery, p. 187.

Case 2.—A little girl of about 9 years, fell out of a swinging hammock, and struck her mouth against the edge of a piazza floor.

The inferior lip was lacerated horizontally, the two superior centrals and temporary cuspidatus of the right side entirely dislocated. About one hour after the accident they were replaced, and retained by silk ligatures.

Subsequently they were pressed into their sockets and retained *in situ*, by means of a semicircular piece of soft wood, cut to receive the incisive edges; which piece was secured to the back teeth by ligatures. The wound in the lip was duly dressed. Severe inflammation of the gums followed, also of the lip and soft parts of the mouth.

The replaced teeth for six weeks continued very irritable and sore. No nourishment could be taken excepting liquids; the patient gradually became weaker in consequence, and subject to syncope, &c., so that, at the expiration of this time it was deemed imperative to remove them. So soon as this was done, she steadily regained her health and strength. I saw her again about four years after the accident. The permanent cuspidati had made their appearance; the lateral incisors approximated toward each other so as to occupy about one-half the space made by the loss of the centrals. By the time she arrives at adult age, it will probably be so nearly filled that she will not require any artificial substitute.

The maxillary arch will be contracted somewhat, and the mouth look *narrow;* but as she fortunately has a long upper lip and naturally exposes the teeth but little, this will scarcely be a source of regret.

The error in replacing the luxated teeth in her case was, in the fact that *the roots were not fully formed*, and however they might have united with their sockets in a membraneous connection, the progress of *growth*, of course, was forever destroyed.

Teeth so *porous* as those of persons of her age and delicate constitution are, would soon have assumed a dark hue—a pink, purple, or chocolate color, which would have greatly marred, if not entirely destroyed their beauty.

The most favorable cases for replacement I should deem to be those of adult, healthy persons—not subject to mucous, glandular, tubercular, or inflammatory disease; having hard teeth, firm gums, and healthy saliva. The teeth—the ten anterior of both jaws; or even a molar, if luxated by accident, as in case of attempt to extract another. I have met with such cases which were doing well several years after the replacement.

In replacing luxated teeth, therefore, I should be governed by four primary considerations:

1. That there should not be extensive fracture or splintering of the alveolus.

2. That the periosteum of the luxated tooth be in a healthy condition—which also implies the vitality of the *tooth* up to the time of the accident—that it be not *lacerated* while out of the socket.

3. That it be replanted within one hour.

4. That it be immovably and uninterruptedly retained *in situ* until the ruptured parts unite by healing.

The proportion of *favorable* cases it must be admitted, is very small. The usual proposed and adopted plans of effecting this last important object are generally ineffectual. Simple ligatures of silk, or fine wire, secured to the adjoining teeth, are usually recommended.

*Berdmore* (the true *father of English dentistry)* advised, for a replaced tooth of a person "advanced in years," to be first drilled through sideways near the gum, to admit and retain the ligature, so as to "secure the tooth more perfectly." Also, to cut off the end of the root; and, in close dentures, to file off the sides of the crown before replacing.*

But I believe the less meddling with the luxated tooth before replacing it, the better. If, however, a luxated tooth—a superior incisor for instance, had a decayed cavity in it, and it were *convenient* to plug it immediately after such accident, it might be better carefully and expeditiously to do so before re-

* "*Treat. on the Disorders and Deformities of the Teeth and Gums.*" Balt. Ed. p. 34.

placing it, than to risk the effect of subsequent disturbance by pressure, &c.*

*Securing the replaced tooth.* The simple thread or wire ligature thrown around it, and secured to an adjoining one, cannot be made sufficiently steady to give *repose* to the replanted tooth, and allow it to become fixed—neither does it provide against concussions and shocks from the natural occlusion of the jaws. My treatment in such case would be first—to take an impression of the mouth in very *soft* warm wax in the usual manner. Next—after syringing out the sockets with warm water and carefully washing the tooth in the same, press it in its socket and secure it by a silk ligature. Then proceed to strike up a metallic *plate,* and adjust clasps to it properly, as for sustaining one of artificial teeth.

Next adjust and solder on a concave cylindrical *backing,* to embrace and support the replanted tooth. In this backing or stay, holes may be punched to receive the silken ligature which is to pass around the tooth and be tied. Now, to guard against any concussions or shocks from occlusion of the jaws—the *plate* will be the best means of sustaining pieces of India rubber, or metallic sacks, or thimbles, to interpose between the antagonistic molars, and sustain all the pressure arising therefrom. The plate and apparatus may be carefully removed occasionally as necessary, for the purpose of cleansing, and the ligature renewed. By such means the tooth may be kept perfectly steady until the membranes shall unite by the "first intention." Un-

* A popular *superstition* is yet prevalent that a foreign human tooth, however long removed, or even an *artificial one,* may be "fixed in the jaw-bone" and comfortably retained there! It is only necessary, *it is believed,* to insert the substitute immediately in the vacated socket, and there keep it until the gum closes around it, when it is "fixed!" I have been repeatedly entertained with glowing accounts of this "successful" miracle!

Some ten years ago I extracted a superior bicuspid for a man who insisted on having it replaced on his own plan despite my admonitions to the contrary. He directed me to shorten the root by filing—to scrape the periosteum entirely off. He then pushed it back into its socket, tied up his face and left "in hopes." I learned next day that he had passed a sleepless night, and in the morning was compelled to pick out the *thorn in his flesh.* No enlightened person could reasonably hope for success in such case.

less this end is accomplished, the operation may be regarded as a failure.

To this end of speedy union, it is important to subdue as much as possible all general and local inflammatory symptoms—as by bleeding, purging, astringent lotions, &c. The diet should be in the form of broths, soups, and soft food, to avoid irritation in mastication.

Those wishing to inform themselves more particularly on the subjects of accidental luxation of the teeth, fractures of the alveolus, maxilla, &c., are referred to the following authors and works:

*Bell.*—"Anat. Phys. and Dis. of the Teeth." P. 177.

*Bond.*—"Dent. Med." 1st ed. p. 216.

*Desirabode.*—"Complete Elements," &c., Balt. ed. p. 200.

*Fox.*—"Nat. Hist. and Dis. of the Teeth," London ed. p. 58.

*Gariot.*—"Dis. of the Mouth," Am. ed. pp. 83, 98-9.

*Goddard.*—"Anat. Phys. and Path. of the Hum. Teeth," chap. 8, ¶ 251.

*Hunter.*—"Nat. Hist. and Dis. of the Hum. Teeth," Phil. ed. p. 97., N. Y. ed. part ii, p. 36.

*Harris.*—"Princ. and Prac. Dent. Surg." 4th ed. p. 410. "Dic. Dent. Sci.," art. "Fractures."

*Jourdain.*—"Dis. and Surg. Op. of the Mouth," Balt. ed. pp. 280-4.

*Lefoulon.*—"Nouveau Traite Theorique et Pratique de l'art du Dentiste," Paris ed. pp. 267-272.

*Maury.*—"Dental Art." Phil. ed. p. 95.

*Tomes.*—"Lectures on Dent. Phys. and Surg." London ed. p. 183. &c. &c.

